# Elevated levels of sIL-2R, TNF-α and hs-CRP are independent risk factors for post percutaneous coronary intervention coronary slow flow in patients with non-ST segment elevation acute coronary syndrome

**DOI:** 10.1007/s10554-022-02529-8

**Published:** 2022-02-19

**Authors:** Cheng Wang, Yan Wu, Yang Su, Bin Mao, Yihong Luo, Yexiang Yan, Kun Hu, Yi Lu, Wenliang Che, Minying Wan

**Affiliations:** 1 Department of Cardiology, Chongming Branch, Shanghai Tenth People’s Hospital, Tongji University School of Medicine, 66 East Xiangyang Road, Chongming, Shanghai China; 2 Department of Cardiology, Shanghai Putuo District Liqun Hospital, Shanghai, 200333 China; 3 Department of Cardiology, Shanghai Tenth People’s Hospital, Shanghai, 200072 China

**Keywords:** Non-ST segment elevation acute coronary syndrome (NSTE-ACS), Coronary slow flow (CSF), Percutaneous coronary intervention (PCI), Inflammatory cytokines

## Abstract

To evaluate the association between circulating levels of inflammatory cytokines and the occurrence of post-percutaneous coronary intervention (PCI) coronary slow flow (CSF) in patients with non-ST segment elevation acute coronary syndrome (NSTE-ACS). CSF after PCI commonly occurs and implies poor outcomes, while the determinants of post-PCI CSF in patients with NSTE-ACS remain controversial. In this multicenter case control study, 176 patients diagnosed with NSTE-ACS and with post-PCI CSF occurred composed of CSF group, while 352 matched NSTE-ACS patients composed control group. Corrected thrombolysis in myocardial infarction frame count (cTFC), circulating levels of inflammatory cytokines and PCI related parameters were analyzed using Logistic regression models. Among 528 patients with median age of 67 (59–76) and male proportion of 65.5%, 176 (35.0%) patients had occurrence of post-PCI CSF defined as cTFC ≥ 24. Patients with CSF presented more intense inflammatory activity revealed by higher levels of white blood cell, high-sensitivity C-reactive protein (hs-CRP), interleukin-1ß (IL-1ß), soluble IL-2 receptor (sIL-2R), IL-6, IL-8, IL-10 and tumor necrosis factor-α (TNF-α), while PCI related parameters were comparable. Correlation analysis showed cTFC was positively correlated with those inflammatory cytokines. Logistic regression model indicates that hs-CRP (odds ratio (OR) = 3.038, 95% confidence interval (CI) 1.545–5.975), sIL-2R (OR = 2.103, 95% CI 1.959–4.026) and TNF-α (OR = 3.708, 95% CI 1.426–9.641) were valuable predictors for CSF occurrence. Elevated circulating levels of inflammatory cytokine including hs-CRP, sIL-2R and TNF-α rather than PCI related parameters could predict post-PCI CSF in patients with NSTE-ACS.

**Supplementary Information:**

The online version contains supplementary material available at 10.1007/s10554-022-02529-8.

## Introduction

Coronary slow flow (CSF) is a common phenomenon in acute coronary syndrome (ACS) patients, presenting as significant deceleration of coronary blood flow. Compared with non-CSF, patients with CSF have a higher incidence of major adverse cardiovascular events (MACE) during the follow-up period. Furthermore, post-percutaneous coronary intervention (post-PCI) CSF should draw additional attention due to the occurrence of microvascular thrombosis and/or endothelial dysfunction that contributes to worse outcomes [1]. According to literatures, the incidence of post-PCI CSF is 14–25% in patients with ST-segment elevation myocardial infarction (STEMI) [2]. Previous studies among patients with STEMI have showed that hyperglycemia, longer reperfusion time, higher stent to vessel diameter ratio and heavier thrombus burden independently predict post-PCI CSF [3]. However, plaque morphology of non-ST segment elevation acute coronary syndrome (NSTE-ACS) differs from that of STEMI, which is characterized by thicker fibrous cap, more calcified nodules, more intensively diffused atherosclerosis and milder thrombus burden [4]. Therefore, the risk factors for post-PCI CSF in patients with NSTE-ACS could be disparate from those for STEMI and should be independently evaluated. Previous studies have showed that determinants of post-PCI CSF in patients with NSTE-ACS are multifarious, including metabolic indices as BMI, lifestyle factors as smoking status [5], serological biomarkers such as biomarkers of cardiac injury [6], fibrinogen, ischemic modified albumin [7], homocysteine [8], soluble adhesion molecules [9] and choline [10]. Throughout published literatures, determinants of post-PCI CSF in patients with NSTE-ACS has remained controversial.

Inflammatory response plays a crucial role in the development of atherosclerosis, especially in culprit or non-culprit plaques in patients with ACS [11]. Mounting evidences have supported that inflammation is a central pathogenetic process of the progression of atherosclerosis, formation of unstable plaque as well as plaque rupture [12]. The more intense inflammatory response is, the more easily atheroma eruption and microvascular spasm occur. While circulating level of cytokines could indicate the intensity of inflammation, especially TNF-α and interleukins that participate in endothelial dysfunction, inflammatory invasion and development of unstable atherosclerotic plaque [13–17]. Therefore, we suppose that circulating inflammatory cytokines could act as important predictors for CSF phenomenon. The present retrospective cohort study is designed to reveal the expression patterns of inflammatory cytokines in patients with NSTE-ACS, and validate the predictive value of these cytokines for post-PCI CSF phenomenon.

## Materials and methods

### Study population

In this multi-center case–control study, data of patients who diagnosed with NSTE-ACS and underwent PCI procedure were collected from 3 centers including Shanghai Tenth People’s Hospital, Chongming Branch of Shanghai Tenth People’s Hospital and Putuo District Liqun Hospital from February 2014 to January 2016. The study was approved by the Ethics Committee of Shanghai Tenth People’s Hospital (SHSY-IEC-4.1/20-139/01), the Ethics Committee of Chongming Branch of Shanghai Tenth People’s Hospital (SYCM-YJKT-20-0814/01) and the Ethics Committee of Putuo District Liqun Hospital (RT-202013). Written informed consent for blood sample detection and medical treatment was acquired from each patient on admission.

The inclusion criteria were (1) ≥ 18 years old, (2) patients diagnosed as NSTE-ACS according to 2015 ESC guideline [18], and (3) patients required to undergo revascularization according to 2014 ESC/EACTS guideline on myocardial revascularization [19]. The exclusion criteria were (1) severe liver or kidney diseases, (2) trauma, infection and surgery within past three months.

CSF group (case group) included 176 NSTE-ACS patients with the occurrence of post-PCI CSF, excluding 46 patients received peripheral and/or intracoronary injection of IIb/IIIa inhibitor (tirofiban), 26 patients with insufficient quality of angiographic images and 18 patients with unqualified blood sample. While 352 patients diagnosed with NSTE-ACS and underwent revascularization but without post-PCI CSF occurred were included as control group that was 1:2 matched. A loading dose of antiplatelet drugs (either aspirin 300 mg + clopidogrel 300 mg or aspirin 300 mg + ticagrelor 180 mg) was given to the patients before PCI if the patient hasn’t taken any antiplatelet drug before.

## Data collection

Blood samples were obtained for routine lab tests and specific inflammatory cytokines detection within 24 h since admission and before any treatment including medication and primary PCI. Soluble interleukin-2 receptor (sIL-2R), interleukin-8 (IL-8), interleukin-10 (IL-10) and tumor necrosis factor-α (TNF-α) were measured using ELISA kits (Xinyu Biological Technology Co., Ltd. Shanghai, China). High-sensitivity C-reactive protein (hs-CRP) was measured by a Roche Tina-quant immuno-turbidimetric assay (Roche Diagnostics). Interleukin-1β (IL-1β) and interleukin-6 (IL-6) was measured using an EV3513 cytokine biochip array (Randox Laboratories, Crumlin, UK) and competitive chemiluminescence immunoassays (Randox Laboratories, Crumlin, UK).

Demographic information, physical examination, medical history, electrocardiogram and echocardiography were acquired from electronic records. Film and video data of angiography and PCI were collected for analysis and reviewed by two proficient investigators individually. Measurement of imageological data right after revascularization and before any medication (nitrates, adenosine, calcium antagonist or IIb/IIIa antagonist) was accomplished in order to evaluate the occurrence of CSF.

## Images measurements

All coronary angiography evaluation after PCI was performed using 5F catheter, and images were obtained at the rate of 30 frames per second. Lesion type was classified according to previous study [20]. Haziness was defined as the presence of inhomogeneous contrast and/or indistinct vessel borders and filling defect was defined as the presence of intraluminal region with no filling of contrast [21]. Representative images were displayed in Supplementary Fig. 1. In the situation of multivessel disease, the coronary artery with the most severe lesion was selected for CSF evaluation. When more than one stent was needed for the candidate vessel, the stent diameter was defined as the mean diameter of stents measured after post-dilation and the stent length was the sum of length of implanted stents. Sd/RVd ratio was calculated by the ratio of stent diameter and reference vessel diameter, which assessed the effectiveness of stent implantation [22]. Coronary stenosis was measured using quantitative coronary angiography (QCA) method performed on digital subtraction angiography (DSA) workstation (Siemens, German). Quantitative flow ratio (QFR) after revascularization was measured using AngioPlus (Pulse Medical Imaging Technology, Shanghai, China) based on two different angiographic image views with angel ≥ 25°. CSF was defined as corrected thrombolysis in myocardial infarction (TIMI) frame count (cTFC) ≥ 24 [23]. The cTFC of left anterior descending (LAD) artery was divided by 1.7 to generate an adjusted value due to the longer anatomic length than the other two major arteries. Total Syntax score for each participant was calculated using the SYNTAX score calculator (version 2.28, www.syntaxscore.com).

### Statistical analysis

Statistical analysis was conducted using SPSS 22.0 (IBM Inc., USA). Continuous variables were presented as mean ± standard deviation (SD) or median with interquartile range (IQR), and compared using 2-sample t-test if they conform normal distribution or Mann–Whitney *U*-test if not. Dichotomous variables were presented as proportion and compared using Fisher exact test or χ^2^ test.

Spearman correlation analysis was conducted to explore the association between the cTFC and measured variables. Receiver operator characteristics (ROC) analysis was conducted to evaluate the generate cut-off value of inflammatory cytokines. Furthermore, univariate and multivariate Logistic regression models were built to explore risk factors of CSF. Inflammatory cytokines were incorporated in continuous or binary forms in the multivariate model respectively. The multivariate Logistic model was adjusted for gender, age, fasting blood glucose (FBG), diastolic blood pressure (DBP), heart rate (HR), cardiac troponin T (cTnT), total cholesterol (TC), high density lipoprotein (HDL), stent length and post dilation pressure. All tests were two-sided and p < 0.05 was considered statistically significant.

## Patient and public involvement

The patients, the public or any third parties were not involved in the design, conduct, reporting or dissemination of our research.

## Results

### Baseline clinical characteristics of patients with CSF

Overall, 528 patients with 65.5% male and median age of 67 [59–76] were eligible to be finally included. The inflammatory cytokines concentration distribution in overall population was displayed in Supplementary Fig. 2. The demographic feature and medical history were comparable between CSF and control groups (Table 1). In CSF group, patients displayed lower level of hemoglobin (Hb), and higher level of white blood cell count (WBC), N‑terminal pro B‑type natriuretic peptide (NT-proBNP), total cholesterol (TC), fast blood glucose (FBG), high sensitivity C-reactive protein (hs-CRP), interleukin-1β (IL-1β), soluble interleukin-2 receptor (sIL-2R), interleukin-6 (IL-6), interleukin-8 (IL-8), interleukin-10 (IL-10) and tumor necrosis factor-α (TNF-α). Likewise, there are no difference in medical history and antiaggregant loading drugs using between groups (Supplementary Table 1). This probably indicate that patients with CSF occurrence after PCI usually had metabolic disturbance and more active inflammatory responses.

**Table 1:** Baseline characteristics and inflammatory cytokine levels in subgroups

	Overall (N = 528)	Non-CSF (N = 352)	CSF (N = 176)	P-value
Age (years)	67 (59–76)	67 (60–76)	67 (59–76)	0.812
Male/female (n/n)	346/182	214/138	132/44	0.093
HR (bpm)	70 (61–75)	68 (61–75)	71 (65–76)	0.109
SBP (mmHg)	130 (120–143)	128 (119–141)	132 (121–143)	0.146
DBP (mmHg)	73 (62–87)	76 (63–88)	69 (61–85)	0.116
DM, n (%)	156 (29.5)	100 (28.3)	56 (31.8)	0.768
HTN, n (%)	334 (63.3)	212 (60.2)	122 (69.3)	0.322
TC (mmol/l)	3.19 (1.55–4.36)	2.85 (1.24–3.97)	3.43 (1.90–4.59)	0.013*
TG (mmol/l)	1.73 (1.11–2.43)	1.48 (1.08–2.47)	1.85 (1.36–2.42)	0.209
HDL (mmol/l)	1.21 (1.02–1.4)	1.27 (1.02–1.45)	1.185 (1.04–1.35)	0.140
LDL (mmol/l)	2.33 ± 0.77	2.38 ± 0.78	2.24 ± 0.75	0.205
LP(a) (mg/dl)	18.9 (11.4–35.8)	19.9 (11.9–35.1)	17.2 (9.9–33.4)	0.143
WBC (/nl)	6.8 (5.7–8.2)	6.4 (5.6–7.7)	7.4 (5.9–8.8)	0.023*
Hb (g/l)	131.6 ± 16.8	133.4 ± 14.4	128.4 ± 20.1	0.036*
FBG (mmol/l)	4.9 (4.2–5.7)	4.6 (3.7–5.6)	5.0 (4.5–5.8)	0.019*
HbA1c (%)	6 (5.6–6.6)	5.9 (5.6–6.4)	6.2 (5.7–7.4)	0.050
cTnT (ng/ml)	17.9 (2.1–36.3)	19.0 (2.9–33.8)	9.0 (1.7–41.6)	0.470
CK-MB (ng/ml)	10.1 (1.8–28.4)	2.9 (1.5–25.2)	21.9 (4.1–37.3)	< 0.001
NT-proBNP (mmol/l)	135.8 (60.4–552.6)	122.5 (60.4–400.6)	202.6 (61.9–974.0)	0.028*
eGFR (ml/(min*1.73m^2^))	96.0 ± 32.4	99.0 ± 31.9	91.1 ± 33.4	0.095
BUN (mmol/l)	5.8 (4.8–7)	5.9 (4.8–6.8)	5.7 (4.9–7.5)	0.302
UA (μmol/l)	326.9 ± 100.8	324.5 ± 95.7	331.0 ± 109.7	0.656
D-dimer (mg/l)	0.11 (0.07–0.17)	0.11 (0.07–0.17)	0.11 (0.08–0.19)	0.438
hs-CRP (mg/l)	2.3 (1.1–5.9)	3.02 (2.3–3.2)	3.1 (3.0–8.4)	< 0.001*
IL-1β (pg/ml)	0.63 (0.40–1.51)	0.53 (0.40–1.28)	1.21 (0.60–2.12)	0.001*
sIL-2R (U/ml)	437.0 (351.0–534.5)	416.0 (343.5–510.0)	468.5 (371.8–562.0)	0.012*
IL-6 (pg/ml)	4.01 (2.56–7.71)	3.44 (2.47–6.17)	5.77 (3.32–9.71)	0.002*
IL-8 (pg/ml)	27.7 (14.3–75.9)	22.5 (12.1–55.3)	52.7 (18.8–105.0)	< 0.001*
IL-10 (pg/ml)	1.3 (0.9–2.0)	1.2 (0.8–1.5)	1.7 (1.2–4.0)	0.003*
TNF-α (pg/ml)	30.3 (16.6–67.5)	24.1 (14.3–52.5)	50.3 (28.1–88.7)	< 0.001*

Continuous variables are presented as the mean ± standard deviation if they conform to a normal distribution, and otherwise as the median with interquartile range. Categorical variables are presented as n (%)

*CSF* coronary slow flow, *HR* heart rate, *SBP* systolic blood pressure, *DBP* diastolic blood pressure, *DM* diabetes mellitus, *HTN* hypertension, *TC* total cholesterol, *TG* total triglyceride, *HDL‑C* high‑density lipoprotein cholesterol, *LDL‑C* low‑density lipoprotein cholesterol, *LP(a)* lipoprotein (a), *WBC* white blood cells, *Hb* hemoglobin, *FBG* fast blood glucose, *HbA1C* glycated hemoglobin A1C, *cTNT* cardiac troponin T, *CK-MB* creatinine kinase-myocardial band, *NT‑proBNP* N‑terminal pro B‑type natriuretic peptide, *eGFR* estimated glomerular filtration rate, *BUN* blood urea nitrogen, *UA* uric acid, *hs-CRP* high sensitivity C‑reactive protein, *IL-1β* interleukin-1β, *sIL-2R* soluble interleukin-2 receptor, *IL-6* interleukin-6, *IL-8* interleukin-8, *IL-10* interleukin-10, *TNF-α* tumor necrosis factor-α

*P value < 0.05. There is significant difference between groups

## Higher inflammatory level appeared in CSF patients subdivided by culprit vessels

**Fig. 1:**
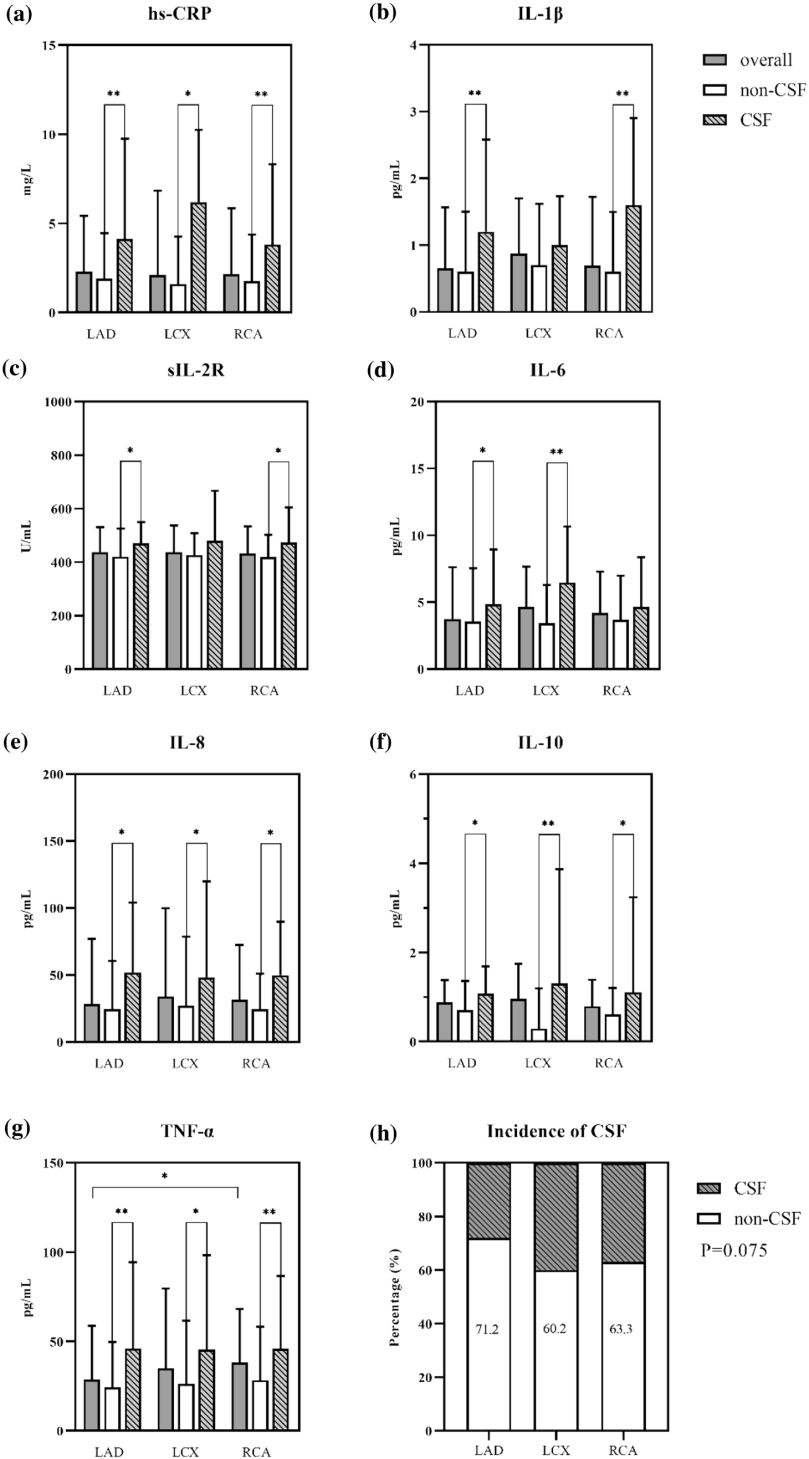
Difference in expression level of inflammatory cytokines and incidence of CSF among patients grouped by culprit artery. *Indicates P value < 0.05, **indicates P value < 0.01. We performed comparisons of **a** inflammatory cytokine levels between CSF patients and non-CSF patients in each culprit vessel, **b** inflammatory cytokine levels of CSF patients among different culprit vessels, and **c** overall levels of inflammatory cytokine among patients with different culprit vessels

The expression level of each cytokine was further explored according to culprit vessels (Fig. 1). Generally, overall cytokines level had no significant difference among 3 culprit vessels except the level of TNF-α was lower in subgroup of LAD compared with that of RCA. While patients with CSF had a higher level of inflammatory cytokine than non-CSF patients within each culprit vessel subgroup, except IL-1β level and sIL-2R in LCX subgroup, and IL-6 in RCA subgroup showed no significant difference between CSF and non-CSF patients. Totally, most of the inflammatory cytokines present higher level in CSF patients regardless of culprit vessels, and the incidence of CSF was comparable among coronaries.

## Angiographic findings and PCI related parameters

The peri-procedure data were analyzed between groups as displayed in Table 2. The median cTFC of patients with CSF and non-CSF was 27 [25–30] and 19 [16–21] respectively. CSF occurrence presented comparably among 3 culprit vessels. The severity and complexity of the coronary lesion between groups were similar, measured by number of diseased vessels, syntax score, lesion type and calcified lesion. While the appearance of angiographic haziness and filling defect in CSF patients was significantly higher (13.6% vs. 6.5%, P-value = 0.001; 4.0% vs. 0.6%, P-value = 0.013, respectively). Focusing on the relevant parameters in PCI procedure, there were no significant difference between groups on stent length, the proportion of single-stent treatment, post-dilation pressure and post-dilation counts. Besides, the geometric and functional results after revascularization was comparable, indicated by Sd/RVd ratio and post-PCI QFR. As Table 2 presented, there were no significant differences in lesion feature and procedure details between CSF and control groups except haziness and filling defect.

**Table 2:** Comparison of angiographic findings and PCI related parameters

	Overall (N = 528)	Non-CSF (N = 352)	CSF (N = 176)	P-value
*Angiographic findings before revascularization*				
Culprit vessel				0.075
LAD, n (*%*)	267 (50.6)	190 (54.0)	77 (43.8)	
LCX, n (*%*)	103 (19.5)	62 (17.6)	41 (23.3)	
RCA, n (*%*)	158 (29.9)	100 (28.4)	58 (33.0)	
Number of diseased vessels				0.075
1 vessel, n (%)	184 (34.8)	134 (38.1)	50 (28.4)	
2 vessels, n (%)	145 (27.5)	90 (25.6)	55 (31.3)	
3 vessels, n (%)	191 (36.2)	122 (34.7)	69 (39.2)	
Syntax score	19.5 (12.0–26.0)	20.0 (11.5–24.3)	18.0 (13.8–29.1)	0.386
*Culprit lesion feature*				
Lesion type				0.405
A and B1, n (%)	86 (16.3)	54 (15.3)	32 (18.2)	
B2 and C, n (%)	442 (83.7)	298 (84.7)	144 (81.8)	
Bifurcation, n (%)	52 (9.8)	39 (11.1)	13 (7.4)	0.179
Calcified lesion, n (%)	124 (23.5)	76 (21.6)	48 (27.3)	0.147
Haziness, n (%)	47 (8.9)	23 (6.5)	24 (13.6)	0.001*
Filling defect, n (%)	9 (1.7)	2 (0.6)	7 (4.0)	0.013*
cTFC	21 (17–26)	19 (16–21)	27 (25–30)	< 0.001*
*PCI related parameters*				
Sd/RVd ratio	0.86 ± 0.08	0.85 ± 0.08	0.87 ± 0.08	0.169
Stent diameter (mm)	3 (2.75–3.5)	3 (2.75–3.5)	3.25 (2.94–3.5)	0.150
Stent length (mm)	24 (18–33)	24 (18–33)	23 (17–30)	0.428
Single stent, n (%)	179 (82.5)	115 (85.2)	64 (84.2)	0.850
Post-dilation pressure (atm)	14 (11–18)	14 (10–17)	15 (12–19)	0.255
Post-dilation counts (n)	2 (1–4)	2 (1–4)	3 (1–4)	0.219
Post-PCI QFR	1 (0.87–1)	1 (0.88–1)	1 (0.86–1)	0.304

Continuous variables are presented as the mean ± standard deviation if they conform to a normal distribution, and otherwise as the median with interquartile range. Categorical variables are presented as n (%)

*CSF* coronary slow flow, *LAD* left anterior descending artery, *LCX* left circumflex artery, *RCA* right coronary artery, *Sd/RVd ratio* stent diameter/reference vessel diameter, *PCI* percutaneous intervention, *QFR* quantitative flow ratio, *cTFC* corrected thrombolysis in myocardial infarction frame count

*P value < 0.05. There is significant difference between groups

## Correlations between cTFC and inflammatory cytokines

To explore the relevant factors of coronary blood flow velocity presented by cTFC, Spearman correlation analysis were conducted. As Table 3 showed, 8 factors revealed positive correlation with cTFC, including TC (r = 0.160), hs-CRP (r = 0.272), IL-1β (r = 0.200), sIL-2R (r = 0.199), IL-6 (r = 0.215), IL-8 (r = 0.148), IL-10 (r = 0.207) and TNF-α (r = 0.240). However, there was no correlation between cTFC and blood cell counts, glucose metabolism, cardiac condition and PCI related parameters. Hence, the circulating level of these inflammatory cytokines might positively stand for the severity of post-PCI CSF phenomenon.

**Table 3:** cTFC positively correlated with inflammatory cytokines

	r	P value		r	P value
Hb	0.157	0.220	WBC	0.210	0.081
FBG	0.086	0.214	hs-CRP	0.272	< 0.001*
TC	0.160	0.042*	IL-1β	0.200	0.026*
NT-proBNP	0.127	0.066	sIL-2R	0.199	0.004*
Sd/RVd ratio	0.063	0.361	IL-6	0.215	0.004*
Post-PCI QFR	-0.068	0.324	IL-8	0.148	0.032*
Syntax score	0.044	0.524	IL-10	0.207	0.021*
Stent length	0.019	0.781	TNF-α	0.240	< 0.001*

Spearman correlation analysis were performed to test the correlation between corrected thrombolysis in myocardial infarction frame count (cTFC) and blood glucose, blood lipid, lesion severity, stenting procedure and inflammatory factors. cTFC was positively correlated with hs-CRP, IL-1β, sIL-2R, IL-6, IL-8, IL-10, TNF-α and TC

*Hb* hemoglobin, *FBG* fast blood glucose, *TC* total cholesterol, *NT‑proBNP* N‑terminal pro B‑type natriuretic peptide, *Sd/RVd ratio* stent diameter/reference vessel diameter, *PCI* percutaneous intervention, *QFR* quantitative flow ratio, *hs-CRP* high sensitivity C‑reactive protein, *IL-1β* interleukin-1β, *sIL-2R* soluble interleukin-2 receptor, *IL-6* interleukin-6, *IL-8* interleukin-8, *IL-10* interleukin-10, *TNF-α* tumor necrosis factor-α

*P value < 0.05, the correlation is significant

## Inflammatory cytokines act as risk factors of post-PCI CSF

**Fig. 2:**
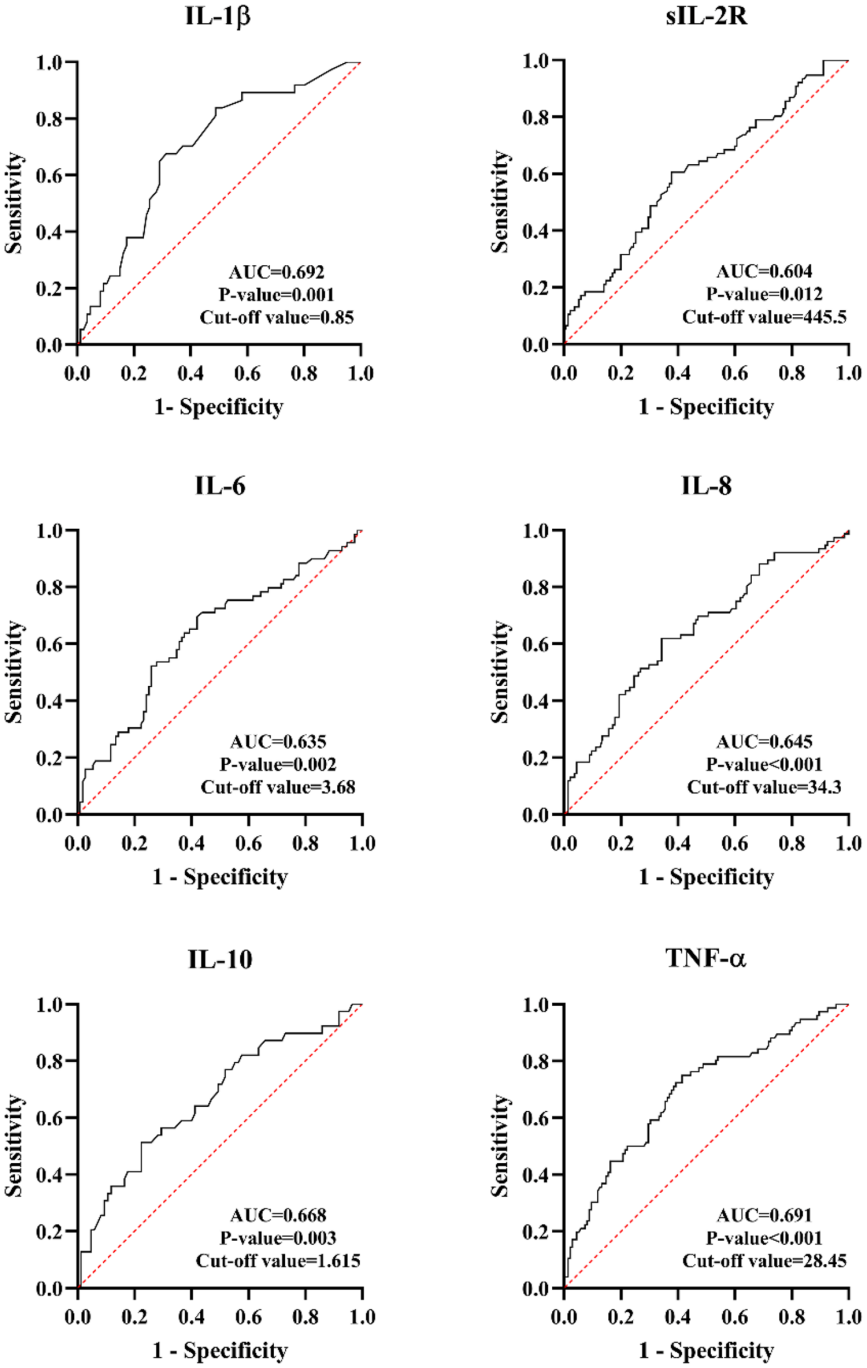
Cut-off values of inflammatory cytokines analyzed by ROC curves. ROC curves were conducted to calculate the cut-off values of each cytokine

**Fig. 3:**
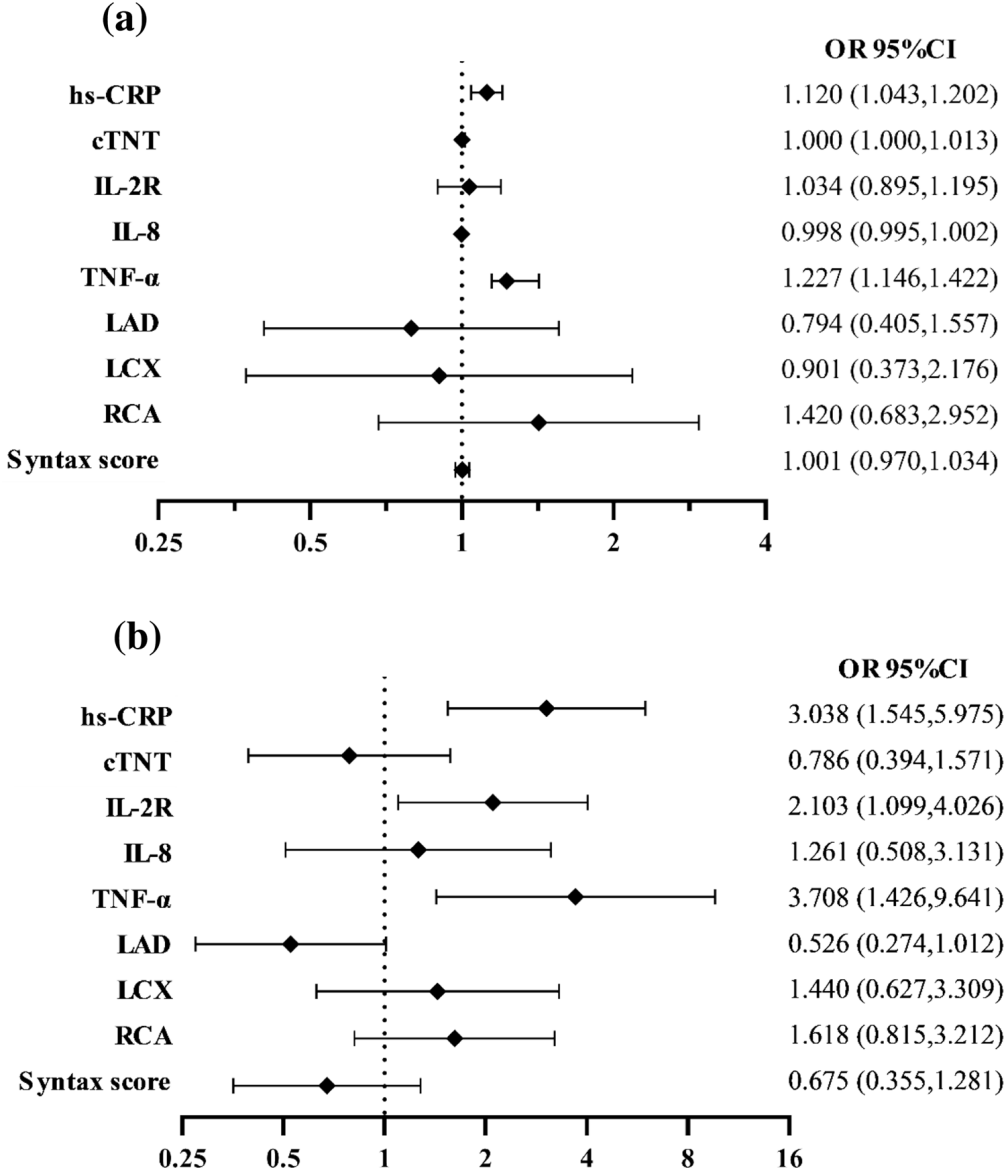
Multivariate Logistic regression models of predictive factors for CSF. Multivariate Logistic regression models of predictive factors for CSF incorporating continuous variables (**a**) and dichotomized variables (**b**). The models were adjusted for gender, age, fast blood glucose, dilated blood pressure, heart rate, cardiac troponin T, total cholesterol, high density lipoprotein, stent length and post dilation pressure. The risk of post-PCI CSF increases 1.120 fold per 1.0 mg/l increment of hs-CRP level and the risk increases 1.227 fold per 10 pg/ml increment of TNF-α (**a**). Continuous variables were dichotomized by cut-off value. The risk of post-PCI CSF increases 3.038 fold in patients with higher level of hs-CRP, the risk increases 2.103 fold in patients with higher level of sIL-2R and the risk increases 3.708 fold in patients with higher level of TNF-α (**b**)

The cutoff values of inflammatory cytokines were calculated from ROC analysis for further variables dichotomy (Fig. 2). To explore potential risk factors of post-PCI CSF, univariate Logistic regression model showed that cTNT and inflammatory cytokines including hs-CRP, sIL-2R and TNF-α were predictor variables of post-PCI CSF. After adjustment for confounding factors, hs-CRP and TNF-α showed predictive value for post-PCI CSF (Fig. 3a). Furthermore, variables of inflammatory cytokines were divided into dichotomous variables by cutoff values. The new multivariate Logistic regression model indicated that higher level than cut-off values of hs-CRP (OR = 3.038, P-value = 0.001), sIL-2R (OR = 2.103, P-value = 0.025) and TNF-α (OR = 3.708, P-value = 0.007) could not only independently predict the occurrence of post-PCI CSF but also presented stronger predictive value (Fig. 3b).

## Discussion

This is the first study comprehensively evaluated the expression pattern of hs-CRP, IL-1ß, sIL-2R, IL-6, IL-8, IL-10 and TNF-α, and explored the predictive value of baseline level of those inflammatory cytokine for post-PCI CSF phenomenon in a NSTE-ACS cohort. Compared with non-CSF, the level of inflammatory cytokines was significantly elevated in patients suffered from CSF after revascularization, indicating the more intense inflammatory response in the population. Besides, the positive correlation between level of inflammatory cytokines and cTFC indicates that those cytokines could reflect the severity of CSF. Notably, it is the level of 3 inflammatory cytokines, hs-CRP, sIL-2R and TNF-α, rather than other factors including metabolic disturbance, severity of myocardium injury, complexity of coronary lesion or PCI related parameters that showed independent predictive value for the occurrence of post-PCI CSF.

Of note, the comparison of angiographic findings shows that patients with CSF have a higher incidence of haziness and filling defect. Previous studies using intracoronary imaging modalities show that haziness and filling defect on angiography could be resulted from miscellaneous causes including plaque rupture, thrombosis and dissection revealed by IVUS or Swiss cheese appearance by OCT [24]. Therefore, angiographic findings alone provide only limited information, which could not comprehensively reveal the feature of underlying lesion. Owning to the limited sample size of our study, the association of haziness/filling defect and CSF needs to be investigated in future studies.

Despite CSF occurred less frequently in patients with NSTE-ACS compared STEMI, this phenomenon should still draw our attention owning to its significant contribution to adverse outcomes [25, 26]. Undoubtedly, it is of great importance to ascertain the predictors for the occurrence of post-PCI CSF. In patients with primary CSF, defined as slow coronary filling in absence of stenosis, cTFC has been confirmed to be positively correlated with multitudes of inflammatory cytokines including hs-CRP, IL-6 [27, 28] and Interferon-γ receptor 1 [29]. In patients with NSTEMI, elevated lipoprotein-associated phospholipase A2 which acts as a vascular specific inflammatory cytokine could predict post-PCI CSF occurrence [30]. Besides, a small amount of study reported that adipocytokines played a protective role in patients with CAD who experienced CSF [31, 32]. In accord with previous researches, our study demonstrated that the inflammatory cytokines were significantly correlated with CSF occurrence after revascularization. However, the correlation coefficients of those cytokines with cTFC are smaller than other studies. It might result from the heterogeneity of population involved. Furthermore, compared with post-PCI CSF, primary CSF phenomenon in previous studies was resulted from local inflammatory response without thrombus disorders, which accounts for the relatively weak relevance in our study.

Accumulated evidences had reported that sustained high level of inflammatory cytokines was strongly linked to heavier thrombus burden and a greater extent of plaque instability [33]. An animal study demonstrated that CSF was resulted from impairment of microvascular integrity induced by altered expression level of IL-6 [34]. Previous experiment showed that another inflammatory cytokine, CRP, was released from vulnerable plaque and could intensify the local inflammatory response [11], which has the potential to predict CSF occurrence. And a large scale of clinical research provided evidence that hs-CRP, a more commonly measured index, is able to predict MACEs in patients with cardiovascular disease [35]. In addition, inflammatory cytokines released through paracrine from epicardial fat tissue would also aggravate CSF via affecting the endothelia function [36]. Furthermore, CSF could be improved by alleviating inflammatory responses regulated by mir-155 [37]. Furthermore. Similarly, in the present study, we have validated that inflammatory response plays an essential role in CSF phenomenon from clinical perspective. While other factors, including elder, hyperglycemia and higher stent to vessel ratio, showed non-significant impact. Collectively, inflammatory cytokines and CSF are inextricable. Patients with NSTE-ACS who had higher baseline level of inflammatory cytokines should be identified as individuals at high risk of developing post-PCI CSF.

Correction and treatment of causative factors are prerequisite for reducing occurrence of CSF during procedure. In PL-ACS registry trial, the mortality of ACS patients with final TIMI 0–2 after PCI was as high as 23.84% through 36-month follow-up [25]. Although traditional medication for improving the CSF/no-reflow followed revascularization were given, studies show that the occurrence of MACE in period of follow-up remained high, as 30.88% from an randomized controlled trial RECOVER [26] and 26.53–39.84% in other clinical researches [38, 39]. Promisingly, emerging researches on anti-inflammatory treatment targeting various cytokines, including anakinra [40], colchicine [41] and canakinumab [42, 43], in patients with coronary atherosclerosis disease (CAD) had made gratifying achievements on improving the prognosis [44]. Anti-inflammatory therapy has been becoming an effective and epochmaking approach that could greatly improve the prognosis of patients with CAD. Based on our results, we speculate that anti-inflammatory therapy locally or systemically during revascularization should reduce the occurrence of CSF in patients with NSTE-ACS. However, additional largescale clinical studies are needed to further assess the utility and safety of the treatment and long-term outcomes.

Besides, the predictor of CSF or no-reflow phenomenon has been under debate. In 2019, Mustafa et al. have published a study that investigated the association of no-reflow phenomenon and serum inflammatory biomarkers in patients with STEMI. They found serum CD40 ligand level, as an indicator platelet activation, could be a predictor of no-reflow phenomenon [45]. While inflammatory biomarkers including hs-CRP and WBC count could not predict no-reflow, which is inconsistent with our results. We believe such disparity could be attributed to the limited sample size of both studies, the difference in studied population and the difference between CSF and no-reflow. Based on current evidences, both inflammation and platelet activation play a role in CSF, and inflammation could interlay with platelet in a complicated mechanism that are yet not well understood. Further well-designed studies with larger sample size are needed to provide stronger evidence, and further researches on the relationship between inflammation and platelet activation are needed to unravel their underlying mechanism.

## Limitations

The study is lack of concise morphological description of plaque which was usually measured using intravascular ultrasound. The mechanism of post-PCI CSF was more complicated than primary CSF due to various contributors. Further study is warranted to comprehensively explain the determinants of post-PCI CSF. Besides, we only validate common inflammatory cytokines, further studies thoroughly detecting inflammatory cytokines are necessitated. In addition, microcirculatory dysfunction has been proposed as a principal mechanism of CSF, which haven’t been assessed in our study. Further researches on exploring the relationship between microcirculatory dysfunction and CSF are warranted.

## Conclusion

Elevated circulating levels of inflammatory cytokine including hs-CRP, sIL-2R and TNF-α rather than differences of PCI related parameters could play a crucial role in predicting post-PCI CSF phenomenon in patients with NSTE-ACS. Our results indicate that anti-inflammatory therapy during revascularization could possibly be an effective prevention for CSF in patients with NSTE-ACS.

### Supplementary Information

Below is the link to the electronic supplementary material.

Supplementary file1 (DOCX 1018 kb)

## Data Availability

The datasets used and/or analyzed during the current study are available from the corresponding author on reasonable request.
