# Supplementary material for: Elevated levels of sIL-2R, TNF-α and hs-CRP are independent risk factors for post percutaneous coronary intervention coronary slow flow in patients with non-ST segment elevation acute coronary syndrome
Source: Int J Cardiovasc Imaging. 2022 Feb 19;38(6):1191–202. doi: 10.1007/s10554-022-02529-8 (PMC11143008; doi:10.1007/s10554-022-02529-8)
Supplement: Supplementary file 1 — Supplementary file1 (DOCX 1018 kb) [file 10554_2022_2529_MOESM1_ESM.docx]

**Supplementary materials**


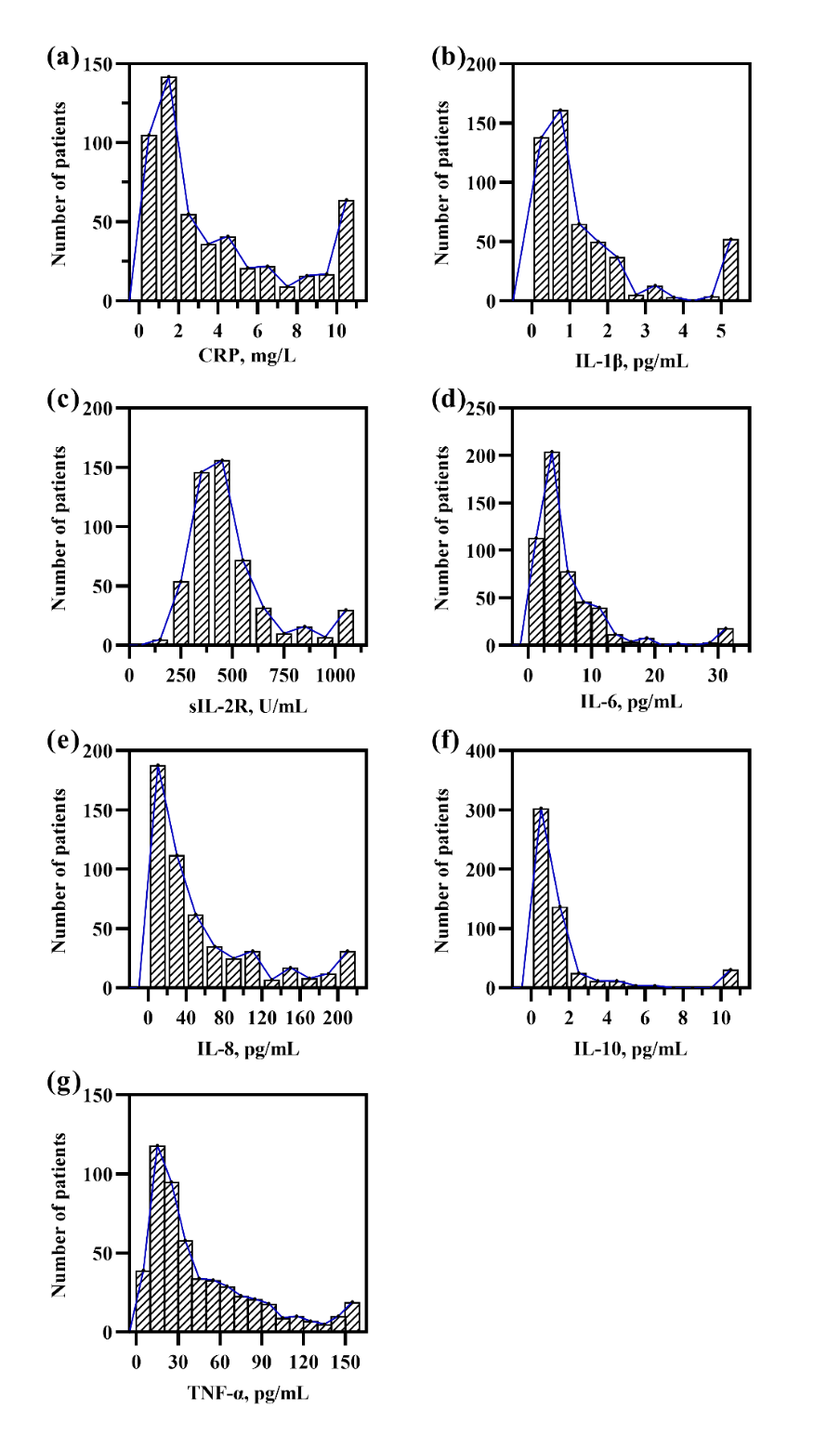


**Supplementary figure 1 Distribution of 7 inflammatory cytokines.** For better display of the distribution, large outliners were incorporated into the last column of each histogram.

**
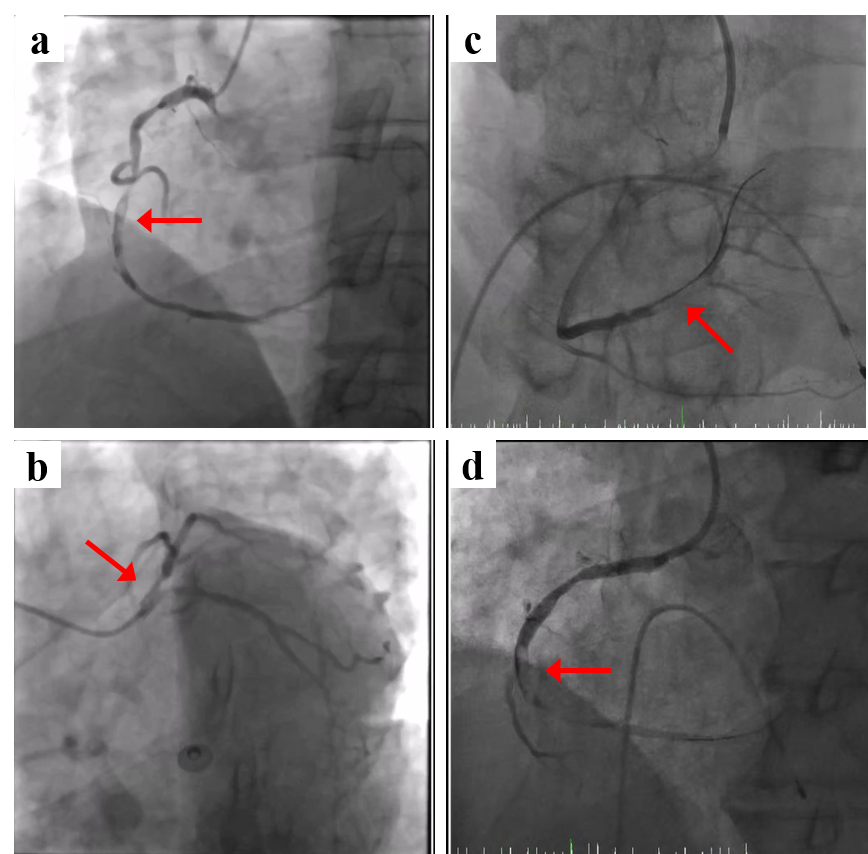
**

**Supplementary figure 2 Representative angiographic findings of haziness and filling defect**. Figure **a** and **b** show haziness where in homogenous contrast can be observed at right coronary artery (**a**) and bifurcation of left coronary artery (**b**) respectively. Figure **c** and **d** show filling defect where no contrast filling can be observed at distal segment (**c**) and middle segment (**d**) of right coronary artery respectively.

**Supplemental table 1 Medical history and antiaggregant agent administration.**

|  | **Overall**  **(N=528)** | **Non-CSF**  **(N=352)** | **CSF**  **(N=176)** | **P-value** |
| --- | --- | --- | --- | --- |
| **Medical history** | | | | |
| CHD, n (%) | 167 (31.6) | 114 (32.4) | 53 (30.1) | 0.597 |
| PCI, n (%) | 63 (11.9) | 36 (10.1) | 27 (15.3) | 0.873 |
| Stroke, n (%) | 45 (8.5) | 26 (7.5) | 13 (9.1) | 0.370 |
| AF, n (%) | 39 (7.4) | 26 (7.5) | 13 (7.4) | 1.000 |
| **History of antiaggregant and anticoagulant therapy** | | | | |
| OAC, n (%) | 17 (3.2) | 13 (3.7) | 4 (2.3) | 0.383 |
| SAPT, n (%) | 99 (18.8) | 67 (19.0) | 32 (18.2) | 0.813 |
| DAPT, n (%) | 31 (5.9) | 22 (6.3) | 9 (5.1) | 0.601 |
| **Antiplatelet loading before PCI** | | | | |
| Aspirin+clopidogrel | 108 (20.5) | 75 (21.3) | 33 (18.8) | 0.492 |
| Aspirin+ticagrelor | 389 (73.7) | 256 (72.7) | 133 (75.6) | 0.485 |
| Tirofiban | 273 (51.7) | 188 (53.4) | 85 (48.3) | 0.268 |

CHD denotes coronary heart disease, PCI percutaneous coronary intervention, AF atrial fibrillation, OAC oral anticoagulants, DAPT double anti-platelet therapy, SAPT single anti-platelet therapy.
